# A Meta-Analysis of Red Yeast Rice: An Effective and Relatively Safe Alternative Approach for Dyslipidemia

**DOI:** 10.1371/journal.pone.0098611

**Published:** 2014-06-04

**Authors:** Yinhua Li, Long Jiang, Zhangrong Jia, Wei Xin, Shiwei Yang, Qiu Yang, Luya Wang

**Affiliations:** Department of Arteriosclerosis, Beijing Institute of Heart, Lung and Blood Vessel Diseases, Beijing Anzhen Hospital, Capital Medical University, Beijing, China; University of Milano, Italy

## Abstract

**Objective:**

To explore whether red yeast rice is a safe and effective alternative approach for dyslipidemia.

**Methods:**

Pubmed, the Cochrane Library, EBSCO host, Chinese VIP Information (VIP), China National Knowledge Infrastructure (CNKI), Wanfang Databases were searched for appropriate articles. Randomized trials of RYR (not including Xuezhikang and Zhibituo) and placebo as control in patients with dyslipidemia were considered. Two authors read all papers and independently extracted all relevant information. The primary outcomes were serum total cholesterol (TC), low-density lipoprotein cholesterol (LDL-C), triglyceride (TG), and high-density lipoprotein cholesterol (HDL-C). The secondary outcomes were increased levels of alanine transaminase, aspartate aminotransferase, creatine kinase, creatinine and fasting blood glucose.

**Results:**

A total of 13 randomized, placebo-controlled trials containing 804 participants were analyzed. Red yeast rice exhibited significant lowering effects on serum TC [WMD = −0.97 (95% CI: −1.13, −0.80) mmol/L, P<0.001], TG [WMD = −0.23 (95% CI: −0.31, −0.14) mmol/L, P<0.001], and LDL-C [WMD = −0.87 (95% CI: −1.03, −0.71) mmol/L, P<0.001] but no significant increasing effect on HDL-C [WMD = 0.08 (95% CI: −0.02, 0.19) mmol/L, P = 0.11] compared with placebo. No serious side effects were reported in all trials.

**Conclusions:**

The meta-analysis suggests that red yeast rice is an effective and relatively safe approach for dyslipidemia. However, further long-term, rigorously designed randomized controlled trials are still warranted before red yeast rice could be recommended to patients with dyslipidemia, especially as an alternative to statins.

## Introduction

Cardiovascular disease (CVD) is the leading cause of mortality worldwide and causes 17 million deaths every year[1]. Hyperlipidemia, particularly increased serum total cholesterol, is an important cardiovascular risk factor and causes an estimated 4.4 million deaths every year worldwide[2]. Data from the Cholesterol Treatment Trialists' Collaborators demonstrated that a 1.0 mmol/l reduction in low-density lipoprotein cholesterol(LDL-C) resulted in a 9% reduction in all-cause mortality and a 25% reduction in major vascular events, even among low-risk patients[3].

The statins (3-hydroxy-3-methylglutaryl coenzyme A (HMG-CoA) reductase inhibitors) are the first line lipid-lowering therapy due to their well-known efficacy for reducing cardiovascular morbidity and mortality[4]. A 2013 Cochrane review corroborated a 25% reduction in cardiovascular disease events and a 14% reduction in all-cause mortality with statin therapy despite an a 18% increase in incident diabetes[5]. However, recently much debate has focused on the side effects of statins including myalgias and muscle weakness, reduced energy, increased fatigue, liver enzyme elevations, worsening hyperglycemia and risk of incident diabetes[6]–[8]. Therefore, a safe and effective alternative approach for dyslipidemia management is needed. Currently, more attention is being paid to alternative therapies such as nutrients and Chinese herbal medicine.

Red Yeast Rice (RYR), which has been used as a dietary supplement and as a herbal medicine in China for centuries, may serve as an option for the treatment of hyperlipidemia[9]. The constituents of RYR include Monacolin K (lovastatin) and other active ingredients that are thought to play a role in the management of hyperlipidemia[10]. Clinical studies suggest that RYR has the potential to reduce serum LDL-C levels by 10% to 33%[11]–[13]. The aim of this meta-analysis is to explore whether RYR (not including Xuezhikang and Zhibituo) is a safe and effective alternative approach for dyslipidemia.

## Methods

The meta-analysis was conducted according to the PRISMA statement (Preferred reporting items for systematic reviews and meta-analyses) [14]. The PRISMA checklist for this meta-analysis is shown at Checklist S1.

### Search Strategy

We searched the following 6 databases up to August 2013 for the identification of trials: the Cochrane Library, Pubmed, EBSCO host, Chinese VIP Information (VIP), China National Knowledge Infrastructure (CNKI), Wanfang Databases. The following search strategy was used: dyslipidemia, hyperlipidemia, hyperlipaemia, hypercholesterolemia, hypertriglyceridemia, hyperlipoproteinemia, cholesterol and red yeast rice, monascus, Monascus purpureus, Cholestin. There was no language restriction in our search strategy. An e-mail should be sent to the corresponding author if the outcomes data are not clear in the studies.

### Inclusion and Exclusion Criteria

Randomized placebo controlled trials were considered. Uncontrolled, nonrandom, crossover trials were excluded, and the duration of the intervention was no less than four weeks. The diagnostic criteria for dyslipidemia complied with at least one of the current or past guidelines or definitions of dyslipidemia. Secondary dyslipidemia and familial hypercholesterolemia, serious heart failure, and liver or kidney diseases were excluded. When outcomes were ambiguous or missing in the article and the author could not be contacted, the trial was excluded.

### Outcome Measures

The primary outcomes were serum total cholesterol (TC), LDL-C, triglyceride (TG), and high-density lipoprotein cholesterol (HDL-C). The secondary outcomes were increased levels of alanine transaminase (ALT), aspartate aminotransferase (AST), creatine kinase (CK), creatinine and fasting blood glucose. Blood lipid and glucose levels were collated in mmol/L. If cholesterol levels (TC, HDL, LDL) or TG levels were published in mg/dL, amounts were divided by a factor of 38.67 for cholesterol and 88.55 for triglycerides to convert to mmol/L. If glucose levels were published in mg/dL, amounts were divided by a factor of 18 to convert to mmol/L. AST, ALT, CK were measured in U/L, creatinine was measured in ummol/L (when it was published in mg/dL, amounts were multiplied by a factor of 88.4 to convert to ummol/L).

### Data Extraction and Quality Assessment

Two reviewers (Li YH, Jiang L) independently extracted the following data: (1) general information: title, author, publication date, literature source, and clinical trial sites; (2) characteristics of the included trials and subjects: sample size, gender, age, interventions of each group, and duration of treatment; and (3) outcomes: the levels of TC, TG, LDL-C, HDL-C, ALT, AST, creatinine, and CK at baseline and after treatment. Disagreements were resolved by consultation or consensus with a third reviewer (Jia ZR). The methodological quality of all included studies were assessed according to the Cochrane risk of bias tool, which include 6 aspects: random sequence generation; allocation concealment; blinding of participants, personnel and outcome assessment; incomplete outcome data; and selective reporting and other sources of bias[15].

### Statistical analysis

Statistical analyses were conducted using Revman 5.2 software provided by the Cochrane Collaboration. Because our outcomes were all continuous outcomes, they were presented as weighted mean difference (WMD) and its 95% confidence intervals (CI). Heterogeneity was assessed using the χ2 test. In addition, the I^2^ statistic was documented to describe the percentage of observed variability across the studies due to heterogeneity rather than chance. Heterogeneity was considered to be significant when I^2^>50%. A fixed effect model was used if no significant heterogeneity of the data existed, whereas a random effect model was used if significant heterogeneity existed (I2>50%). *P*<0.05 was recognized as statistically significant. Random-effects meta-regression was performed using STATA 12.0 (Stata Statistical Software: Release 12, StataCorp LP, College Station, Texas, USA) to assess the association between changes in LDL-C levels and the dose of RYR, geographic area, duration of treatment. The funnel plots were used to explore the publication bias. Subgroup analysis was conducted according to the types of comparisons and trial regions.

## Results

### Trials Description

**Figure 1:**
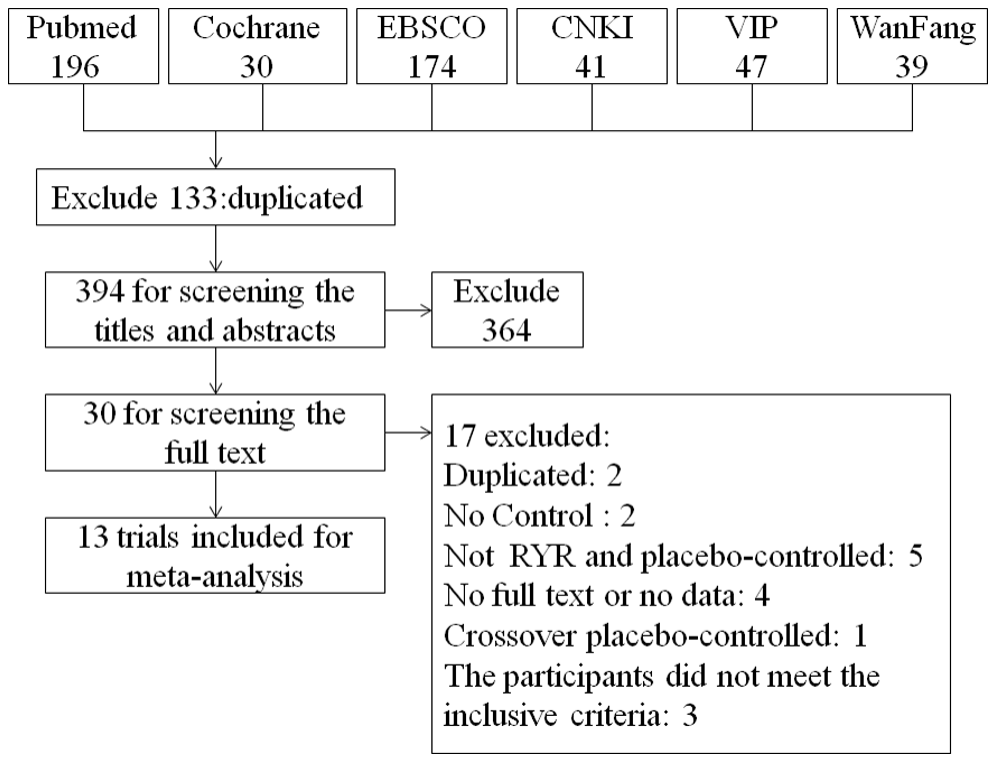
The PRISMA flowchart of trial selection.

A total of 13 randomized controlled trials[16]–[28] from 1999 to 2013 containing 804 participants were included, and all trials were published in English. A total of 527 trials were found after searching the 6 databases mentioned above. After excluding 133 duplicated trials, the remaining 394 were screened for the titles and abstracts. Then, 364 were excluded for different reasons including duplicating, review articles, case reports, comments, non-clinical studies, participants that did not meet the inclusion criteria. Finally, 30 trials remained for screening of the full text, and 17 were excluded: 2 duplicated trials, 2 with no control group, 5 that did not include RYR and a placebo control, 4 with no full text or no data, 1 crossover placebo-controlled trial and 3 with participants that did not meet the inclusion criteria. The entire process of trial selection is shown in Figure 1.

The characteristics of included trials and subjects are listed in Table 1and Table 2. All included trials were placebo-controlled, and the duration of treatment was 4 weeks or longer. Most of included trials were mono-centric except Karl 2012[20] and Yang 2009[25], which were multi-centric. Most of included trials were double-blinded except Marazzi 2011[22], which was single-blinded. TC, TG, LDL-C, HDL-C were reported in most of trials except Huang 2007[27], in which only TC and LDL-C were reported and Karl 2012[20] in which only TC, LDL-C and HDL-C were reported. As presented in Table 1, 9 trials used compounds including red yeast rice (RYR), and 4 used RYR in intervention group, other ingredients and the doses of RYR and lovastatin (monacolin K) per day of every trial were also listed. Among the 13 included trials, 3 trials [18], [20], [25] adopted a three-armed group design. The 3 groups in Barrat 2012[18] were recommended daily dose group (6 tablets, including 1000 mg RYR), the typical dose group (3 tablets, including 500 mg RYR) or the placebo group; all groups were included in this meta-analysis. The 3 groups in Karl 2012[20] were the RYR + Nutrition group, the Nutrition group, and the placebo group; only the RYR + Nutrition group and the placebo group were included in this meta-analysis. The 3 groups in Yang 2009[25] were the RYR + nattokinase group, the mono-nattokinase group, and the placebo group; only the RYR + nattokinase group and the placebo group were included in this meta-analysis.

**Table 1:** Basic characteristics of included trials.

References	clinical trial sites	Sample size(I/C)	Intervention	Control	Doses of RYR per day	Doses of lovastatin	Other ingredients	Duration of treatment
Ogier 2013	France	19/20	Compounds	placebo	500 mg	2 mg	SCdP, artichoke leaf extract	16 weeks
Barrat 2013	France	50/50	Compounds	placebo	500 mg	2 mg	policosanol, artichoke leaf extract	16 weeks
Barrat 2012	France	15/15/15	Compounds	placebo	I1:500 mg/I2:1000 mg	I1:2 mg/I2:4 mg	policosanol, artichoke leaf extract	4 weeks
Lee 2012	Taiwan	54/52	Compounds	placebo	1110 mg	No mention	bitter gourd, chlorella, soybean, licorice	12 weeks
Karl 2012	USA	26/25	Compounds	placebo	1200 mg	4.8 mg	niacin, phytosterol esters, L-carnitine, vitamin C,CoQ-10	8 weeks
Higashikawa 2012	Japan	28/27	Compounds	placebo	900 mg	2 mg	Garlic	12 weeks
Marazzi 2011	Italy	40/40	Compounds	placebo	200 mg	No mention	berberine, policosanol, folic acid, CoQ-10, astaxanthin	12 months
Bogsrud 2010	Norway	22/20	RYR	placebo	1200 mg	7.2 mg	no	16 weeks
Affuso 2010	Italy	25/25	Compounds	placebo	200 mg	3 mg	berberine, policosanols	6 weeks
Yang 2009	Taiwan	19/10	Compounds	placebo	1200 mg	No mention	nattokinase	6 months
Becker 2009	America	31/31	RYR	placebo	3600 mg	6.12 mg	no	24 weeks
Huang 2007	Taiwan	39/40	RYR	placebo	1200 mg	11.4 mg	no	8 weeks
Heber 1999	USA	42/41	RYR	placebo	2400 mg	7.2 mg	no	12 weeks

RYR: Red yeast rice, I:Intervention group, C:Control group, I1: high dose group, I2:low dose group.

**Table 2:** Basic characteristics of included subjects.

References	Male/female	Age(y, I/C)	Baseline TC (mmol/L)	Baseline TG (mmol/L)	Baseline LDL-C (mmol/L)	Baseline HDL-C (mmol/L)	Baseline difference
Ogier 2013	I:6/13, C:5/15	I:50.3±4.8, C:45.7±9.2	I:6.34±0.78, C:6.34±0.78	I:1.02±0.56, C:0.90±0.23	I:4.40±0.52, C:4.40±0.78	I:1.55±0.52, C:1.81±0.52	No
Barrat 2013	I:39/11, C:31/19	I:46.5±11.0, C:47.9±9.6	I:6.10±0.67, C:6.15±0.62	I:0.95±0.40, C:1.02±0.45	I:3.70±0.70, C:3.90±0.67	I:1.53±0.47, C:1.45±0.34	No
Barrat 2012	I1:3/12, I2:4/11, C:7/8	I1:53.4±9.0, I2:50.8±10.8, C:49.1±9.5	I1:6.41±0.67, I2:6.31±0.78, C:6.00±0.65	I1:1.04±0.42, I2:1.04±0.50, C:1.21±0.55	I1:4.09±0.70, I2:3.90±0.57, C:3.98±0.62	I1:1.84±0.39, I2:1.91±0.44, C:1.47±0.31	No
Lee 2012	I:31/21, C:14/30	I:52±10, C:51±10	I:5.4±0.8, C:5.4±0.9	I:2.6±1.1, C:2.3±1.1	I:3.4±0.7, C:3.5±0.8	I:1.1±0.2, C:1.2±0.3	I:more males
Karl 2012	I:12/11, C:3/11	I:60±13, C:63±9	I:5.99±0.22, C:6.4±0.28	No mention	I:3.90±0.19, C:4.36±0.25	I:1.45±0.47, C:1.68±0.70	No
Higashikawa 2012	I:11/17, C:11/16	I:52.0±12.1, C:51.4±11.0	I:5.95±0.87, C:6.10±1.01	I:1.62±0.26, C:1.75±0.36	I:3.93±0.82, C:4.06±1.01	I:1.54±0.36, C:1.49±0.30	No
Marazzi 2011	I:21/19, C:20/20	I:82.45±4.44, C:82.53±4.89	I:6.52±0.60, C:6.54±0.49	I:2.02±0.54, C:2.02±0.56	I:4.45±0.41, C:4.47±0.26	I:1.14±0.31, C:1.14±0.21	No
Bogsrud 2010	No mention	No mention	I:5.69±0.70, C:5.86±1.10	I:1.01±0.60, C:1.29±0.90	I:3.74±0.70, C:4.15±0.90	I:1.62±0.40, C:1.35±0.40	No
Affuso 2010	I:13/12, C:13/12	I:55±8, C:55±7	I:6.60±0.75, C:6.50±0.80	I:1.46±0.82, C:1.67±0.72	I:4.55±0.65, C:4.42±0.57	I:1.50±0.47, C:1.37±0.36	No
Yang 2009	I:8/10, C:4/6	I:54.4±10.4, C:56.3±11.8	I:5.72±0.84, C:5.50±0.98	I:1.70±0.66, C:2.41±1.26	I:3.56±0.79, C:3.28±1.26	I:1.42±0.33, C:1.11±0.24	No
Becker 2009	I:12/19, C:10/21	I:60.5±9.3, C:61.5±8.2	I:6.35±0.79, C:6.37±0.91	I:1.64±0.93, C:1.67±0.87	I:4.23±0.70, C:4.28±0.81	I:1.37±0.31, C:1.33±0.36	No
Huang 2007	I:23/16, C:22/18	I:55.9±8.4, C:59.3±9.6	I:7.28±0.84, C:7.40±1.09	No mention	I:5.2±0.84, C:5.35±1.12	No mention	No
Heber 1999	I:17/20, C:17/20	I:46.3±10.1, C:46.5±9.5	I:6.47±0.78, C:6.59±0.75	I:1.50±0.54, C:1.61±0.52	I:4.47±0.70, C:4.65±0.78	I:1.29±0.34, C:1.19±0.26	No

I: Intervention group, C: Control group, I1: high dose group, I2: low dose group, TC: total cholesterol, TG: triglyceride, LDL-C: low-density lipoprotein cholesterol, HDL-C: high-density lipoprotein cholesterol.

### Quality of the Included Studies

As mentioned above, we assessed the trials according to the Cochrane risk of bias tool in 6 aspects: random sequence generation; allocation concealment; blinding of participants, personnel and assessment; incomplete outcome data; selective reporting; and other sources of bias. All included trials were randomized, placebo-controlled trials, and most of them were double-blinded trials except Marazzi 2011[22], which was single-blinded. Five [17], [18], [20], [21], [26] of the studies reported the methods of random sequence generation. Details of withdrawals and dropouts were reported in all studies. Most of the trials were at low risk of bias for all quality criteria. The details of risks of biases of the included studies are listed in Table S1.

### Effects Estimates of primary outcomes

#### Serum TC level

**Figure 2:**
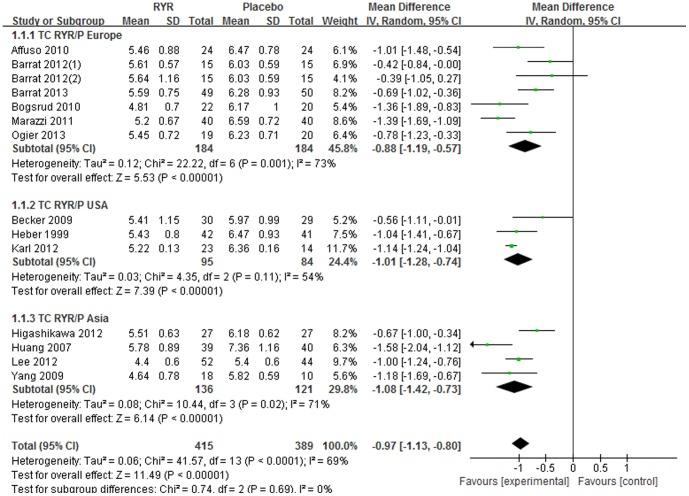
Meta-analysis of Red yeast rice on serum total cholesterol (TC).

Meta-analysis of 13 trials revealed a significant cholesterol-lowering effect of RYR compared with placebo [Total WMD = −0.97 (95% *CI*: −1.13, −0.80) mmol/L, *I^2^* = 69%, *P*<0.001, 13 trials (14 comparisons), n = 804]. The cholesterol-lowering effect was consistent in 3 subgroups divided by different regions: Europe [Subtotal WMD = −0.88 (95% *CI*: −1.19, −0.57) mmol/L, *I^2^* = 73%, *P*<0.001, 6 trials (7 comparisons), n = 368]; USA [Subtotal WMD = −1.01 (95% *CI*: −1.28, −0.74) mmol/L, *I^2^* = 54%, *P*<0.001, 3 trials, n = 179]; and Asia [Subtotal WMD = −1.08 (95% *CI*: −1.42, −0.73) mmol/L, *I^2^* = 71%, *P*<0.001, 4 trials, n = 257]. (Figure 2).

#### Serum TG level

**Figure 3:**
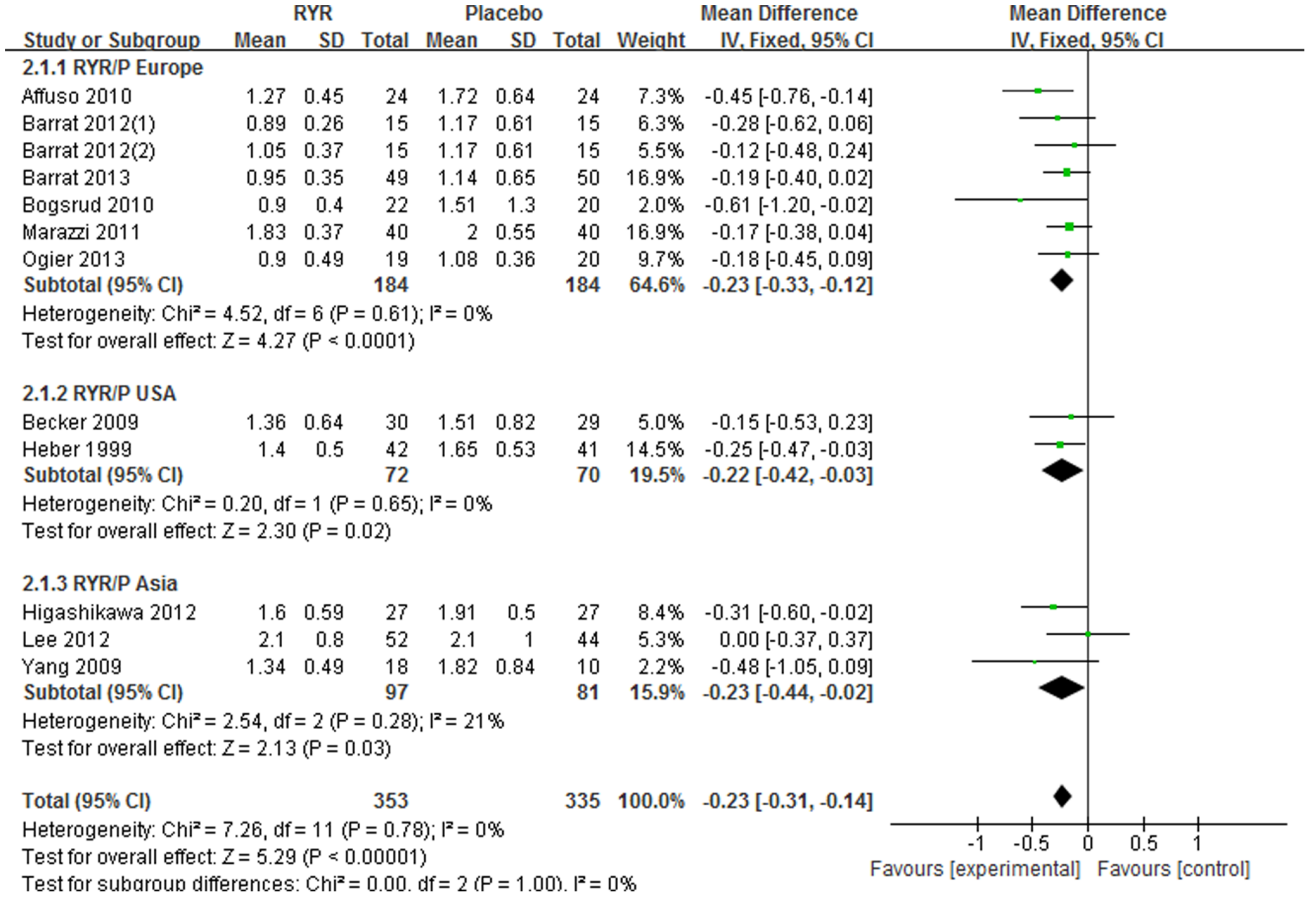
Meta-analysis of Red yeast rice on serum triglyceride (TG).

Most of the studies reported serum TG levels at baseline and after intervention except 2 trials[20], [27]. Compared with placebo, RYR showed a significant triglyceride-lowering effect [Total WMD = −0.23 (95% *CI*: −0.31, −0.14) mmol/L, *I^2^* = 0%, *P*<0.001, 11 trials (12 comparisons), n = 688]. The triglyceride-lowering effect was also significant in 3 subgroups divided by different regions: Europe [Subtotal WMD = −0.23 (95% *CI*: −0.33, −0.12) mmol/L, *I^2^* = 0%, *P*<0.001, 6 trials (7 comparisons), n = 368]; USA [Subtotal WMD = −0.22 (95% *CI*: −0.42, −0.03) mmol/L, *I^2^* = 0%, *P* = 0.02, 2 trials, n = 142]; and Asia [Subtotal WMD = −0.23 (95% *CI*: −0.44, −0.02) mmol/L, *I^2^* = 0%, *P* = 0.03, 3 trials, n = 178]. (Figure 3).

#### Serum LDL-C level

**Figure 4:**
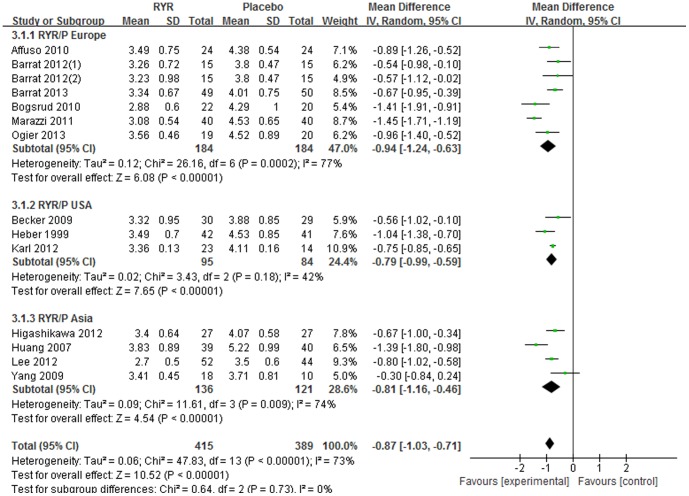
Meta-analysis of Red yeast rice on serum low-density lipoprotein cholesterol (LDL-C).

All studies reported serum LDL-C levels at baseline and after intervention. The meta-analysis indicated that compared with the placebo group, LDL-C level was significantly lower in the intervention group [Total WMD = −0.87 (95% CI: −1.03, −0.71) mmol/L, I2 = 73%, P<0.001, 13 trials (14 comparisons), n = 804]. In addition, when considering the subgroup analysis according to different trial regions, the LDL-C lowering effect was consistent in the 3 subgroups: Europe [Subtotal WMD  = −0.94 (95% CI: −1.24, −0.93) mmol/L, I2 = 77%, P<0.001, 6 trials (7 comparisons), n = 368]; USA [Subtotal WMD  = −0.79 (95% CI: −0.99, −0.59) mmol/L, I2 = 42%, P<0.001, 3 trials, n = 179]; and Asia [Subtotal WMD = −0.81 (95% CI: −1.16, −0.46) mmol/L, I2 = 74%, P<0.001, 4 trials, n = 257]. (Figure 4).

#### Serum HDL-C level

**Figure 5:**
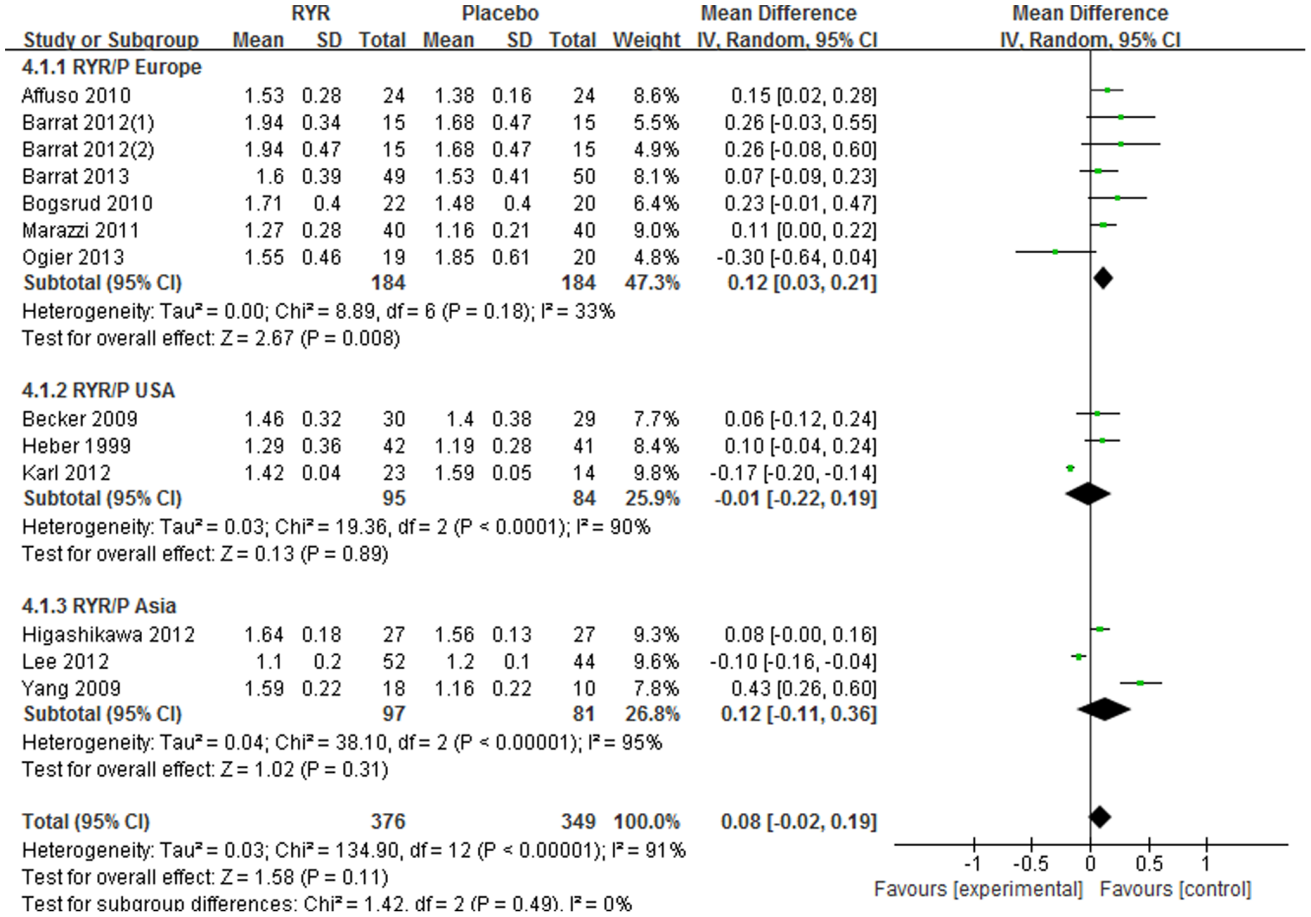
Meta-analysis of Red yeast rice on serum high-density lipoprotein cholesterol (HDL-C).

The effect of RYR on HDL-C level was reported by 12 trials but did not reveal a significant increasing effect compared with the placebo group [Total WMD  = 0.08 (95% *CI*: −0.02, 0.19) mmol/L, *I^2^* = 91%, *P* = 0.11, 12 trials (13 comparisons), n = 725]. However, when divided into 3 subgroups by different regions, the increasing effect of RYR on HDL-C was significant in Europe [Subtotal WMD  = 0.12 (95% *CI*: 0.03, 0.21) mmol/L, *I^2^* = 33%, *P* = 0.008, 6 trials (7 comparisons), n = 368], while not significant in USA [Subtotal WMD  = −0.01 (95% *CI*: −0.22, 0.19) mmol/L, *I^2^* = 90%, *P* = 0.89, 3 trials, n = 179] and Asia [Subtotal WMD  = 0.12 (95% *CI*: −0.11, 0.36) mmol/L, *I^2^* = 95%, *P* = 0.31, 3 trials, n = 178]. (Figure 5).

In addition to RYR, there were other ingredients contained in the included trials, and as berberine may add to the activity of lovastatin being an activator of LDL-receptors, we checked with the trials which contained berberine[22], [24] in the compounds as intervention measure, and we made a meta-analysis of the rest 11 trials, the results showed that their regulating effect of TC [Total WMD  = −0.92 (95% *CI*: −1.10, −0.74) mmol/L, *I^2^* = 70%, *P*<0.001], TG [Total WMD  = −0.17 (95% *CI*: −0.31, −0.03) mmol/L, *I^2^* = 60%, *P = *0.01], LDL-C [Total WMD  = −0.80 (95% *CI*: −0.95, −0.66) mmol/L, *I^2^* = 55%, *P*<0.001] and HDL-C [Total WMD  = 0.07 (95% *CI*: −0.04, 0.19) mmol/L, *I^2^* = 91%, *P* = 0.20] were consistent with that of meta-analysis of the 13 trials.

### Effects estimates of secondary outcomes

We examined the side effects of RYR from 3 aspects: hepatic function (ALT, AST), renal function (creatinine), muscle effects (CK), and fasting blood glucose.

#### Serum ALT level

Meta-analysis of 7 trials[18]–[20], [22], [25], [26], [28] reporting serum ALT level at baseline and after intervention showed that compared with placebo, serum ALT level was significantly higher in the intervention group but within the normal range (0 40 U/L) [Total WMD  = 1.55 (95% *CI*: 0.26, 2.84) U/L, *I^2^* = 0%, *P* = 0.02, 7 trials (8 comparisons), n = 443]. (Figure S1A).

#### Serum AST level

Serum AST level at baseline and after intervention were reported in the same 7 trials as ALT[18]–[20], [22], [25], [26], [28]. Compared with placebo, serum AST level was significantly higher in the intervention group but within the normal range (0 40 U/L) [Total WMD  = 1.47 (95% *CI*: 0.42, 2.51) U/L, *I^2^* = 0%, *P* = 0.006, 7 trials (8 comparisons), n = 443]. (Figure S1B).

#### Serum creatinine level

A total of 4 trials[18]–[20], [25] reported serum creatinine level at baseline and after intervention. There was no statistical significance of serum creatinine level between the intervention group and the placebo group[Total WMD  = 0.33 (95% *CI*: −1.52, 2.18) ummol/L, *I^2^* = 0%, *P* = 0.73, 4 trials (5 comparisons), n = 221]. (Figure S1C).

#### Serum CK level

Analysis of 5 trials[17]–[20], [22] reporting serum CK level indicated no significant CK increasing effect of RYR compared with placebo [Total WMD  = 0.21 (95% *CI*: −21.67, 22.08) U/L, *I^2^* = 87%, *P* = 0.99, 5 trials (6 comparisons), n = 335]. (Figure S1D).

#### Fasting blood glucose level

We made a meta-analysis of 5 trials[18]–[22] reporting fasting blood glucose level, the result indicated no significant glucose increasing effect of RYR compared with placebo [Total WMD  = 0.04 (95% *CI*: −0.11, 0.18) mmol/L, *I^2^* = 68%, *P* = 0.61, 5 trials (6 comparisons), n = 352]. (Figure S1E).

### Meta-regression

The meta-regression analysis on the main end-point (LDL cholesterol reduction) indicated that the effect of RYR in modulating lipid levels was not dependent to the dose of RYR, geographic area, duration of treatment (Table 3).

**Table 3:** Meta-regression analysis.

Variable tested	No. of trials	Coefficient(95% CI)	P value	I-squared_res	Adjusted R-squared
Dose of RYR	13	0.00007 (-0.00017, 0.00031)	0.539	73.18%	-6.33%
Geographic area,	13	0.07052 (-0.16910,-0.31014)	0.533	73.12%	-6.99%
Duration of treatment	13	-0.01059 (-0.02528, 0.00409)	0.142	59.31%	30.19%

RYR: Red yeast rice, No.: number, CI:confidence interval.

### Publication bias

As showed in Figure S2, the trials were distributed averagely in the funnel plot, which indicated no publication bias.

## Discussion

This meta-analysis included 13 random, placebo-controlled trials containing 804 participants. RYR showed significant lowering effects on TC, TG, and LDL-C, but did not show a significant increasing effect on HDL-C compared with the placebo group which was different from the result of the last meta-analysis[13]. It is worth mentioning that when divided into 3 subgroups according to different trial regions, the lowering effect of TC, TG and LDL-C were consistent among the different subgroups, while HDL-C was significantly increased only in European group compared with placebo. No serious side effects were reported in all trials. There were only 2 trials[22], [26] reporting the number of patients with muscle pain in most of the included trials reporting the safety of RYR. However, there was no significant difference in the incidence rate of muscle pain between two groups. Besides, the result of Marazzi 2011[22] suggested a significant improvement of HOMA index in the intervention group [from (1.68±0.63) to (1.51±0.44)] compared to the placebo group [from (1.48±0.63) to (1.48±0.56)], while the changes of the fasting insulin and HbA1c level in the intervention group[fasting insulin: from (7.2±2.4) mcU/mol to (6.9±1.9) mcU/mol, HbA1c: from (5.7±0.32)% to (5.3±0.73)%] were not significantly different from that of the placebo group [fasting insulin: from (6.5±2.4) mcU/mol to (6.6±2.2) mcU/mol, HbA1c: from (5.6±0.26)% to (5.6±0.23)%]. Affuso 2010[24] indicated that there was no significant change in insulin level in the intervention group [from (63.2±36.1) pmol/l to (60.5±39.6) pmol/l] and the placebo group [from (59.7±27.1) pmol/l to (56.3±27.1) pmol/l], and in a subgroup of 11 subjects with insulin resistance (baseline HOMA index>2.6), HOMA index [from (3.3±0.4) to (2.5±1.3)] was significantly reduced, while the QUICKI index [from (0.32±0.005) to (0.34±0.02)] and McAuley index[from (5.73±1) to (6.9±1)] were significantly increased, which indicated enhanced insulin sensitivity. However, the results showed that RYR has a slightly significant increasing effect on serum ALT and AST level compared with placebo which could be tolerated. Therefore, this meta-analysis indicates that RYR is an effective and relatively safe approach for dyslipidemia. Compared with the meta-analysis of RYR for primary hyperlipidemia by Liu JP in 2006[13], our meta-analysis only included RCTs comparing RYR (without Xuezhikang and Zhibituo) with placebo. Besides, most of the included trials in this meta-analysis were published after 2004 and of moderate to high quality. Moreover, we conducted the subgroup analysis according to different trial regions and found that HDL-C was significantly increased only in the Europe group, which may be caused by different baseline lipid levels of different races or other reasons. However, due to the short follow-up time, fewer studies and the small sample size, more long-term RCTs are warranted. In addition, we illustrated the side effects of RYR by visualized and quantized indexes: ALT, AST (hepatic function), creatinine (renal function), CK (muscle effects), fasting blood glucose; the results showed that RYR has no significant increasing effect on serum CK level compared with placebo, which is preferable to statins because of the increasing statin-associated myalgias and CK increasing side effects[29]–[31]. Because of the potential side effects of statins, such as high incidence of muscle pain and potential increasing effect of blood glucose or insulin resistance, the role of nutrition and herbal medicine has been stressed, and it is important to determine whether RYR is a good alternative to statins. The meta-analysis suggested that the lipid modification effects of RYR preparations appeared to be similar to simvastatin, atorvastatin, pravastatin, lovastatin or fluvastatin, but most of the trials used Xuezhikang as intervention[13]. In addition, included studies also demonstrated that RYR was tolerated well in patients who were previously intolerant to statins because of statin-associated myalgia[22], [24], [26], [33], and had no increasing effect of blood glucose[18]–[22], Error! Hyperlink reference not valid. Error! Hyperlink reference not valid. A1C[18], [22] or insulin resistance[22], [24]. There were other studies also showing that RYR was tolerated as well as simvastatin or pravastatin and achieved a comparable reduction of LDL-C, without clinically relevant changes in liver and muscular toxicity markers[32]–[34].

RYR has been used as a dietary supplement and herbal medicine in China for centuries and has also been popularized in the west. A recent study showed that RYR is well tolerated and effective at reducing TC, TG and LDL-C, as well as at increasing HDL-C in Americans with moderate hypercholesterolemia, but those indicators return to the baseline level when the treatment is discontinued[35]. This phenomenon is similar to statins, which could be explained by the main components of RYR (monacolins, which are capable of inhibiting the HMG-CoA reductase enzyme). In addition to lovastatin, most RYR preparations contain other active substances such as Coenzyme Q10 and isoflavones[10], [36]. Moreover, a recent study showed that the oral bioavailability of lovastatin is significantly improved in RYR products as a result of a higher dissolution rate and reduced crystallinity. This indicates that probably the activity of RYR is much higher than predicted based on the very low doses generally given to patients[37]. However, whether RYR preparations should be used as drugs or dietary supplements is still inconclusive. In the US, the FDA recognizes these supplements as drugs when they contain a standardized, specific amount of lovastatin[38]. Despite the lipid regulating benefits, some other positive effects of RYR have also been found in studies, such as improving endothelial function and insulin resistance in patients with mild or moderate hypercholesterolemia, reducing hs-CRP and markers of vascular remodeling in Italian subjects. Moreover, RYR was proved effective, safe and well tolerated in nephrotic dyslipidemia both in adults and children and in familial hypercholesterolemia children[39], [40].

## Limitations

Before recommending the conclusion of this meta-analysis to clinical doctors, we have to note a few limitations in our study. First, although we conducted the subgroup analysis according to different trial regions, there was still heterogeneity of some outcomes (TC, LDL – C, HDL - C), which may be related to different patient populations, doses, and durations of treatment. Second, the outcomes (blood lipids and transaminase) were relatively unstable and were influenced by dietary, life style, and certain medicines, which were most likely not significantly controlled in the included studies. Third, the follow-up time of the included trials is short, and the sample size included for estimation is small; furthermore, some negative trials might not be reported. Forth, the outcomes of this analysis were surrogate indices rather than hard clinical outcomes; therefore the effects of RYR on the clinical endpoints and cardiovascular disease remains to be further studied.

## Conclusions

The results of this meta-analysis suggest that RYR is an effective and relatively safe approach for dyslipidemia, which may be an alternative approach in patients with a history of statin-related adverse effects and could be used for the primary and secondary prevention of coronary heart disease. However, the evidence for RYR as an alternative approach to statins are still insufficient, and more long-term, randomized, double-blinded studies should be conducted. Furthermore, more trials on RYR in patients with cardiovascular disease, diabetes and other diseases combined with dyslipidemia are also warranted.

## Supporting Information

Figure S1 **Analysis of secondary outcomes.** Abbreviations: alanine transaminase (ALT), aspartate aminotransferase (AST), creatine kinase (CK).
(TIF)

Figure S2 **The funnel plots of trials included in the meta-analysis on the effect of Red yeast rice on serum TC (A), TG (B), LDL-C (C), HDL-C (D).** Abbreviations: total cholesterol (TC), triglycerides (TG), low-density lipoprotein cholesterol (LDL-C), and high-density lipoprotein cholesterol (HDL-C).
(TIF)

Table S1 Quality assessment of included trials (Cochrane risk of bias tool).
(DOC)

Checklist S1 **The PRISMA checklist.**
(DOC)
