# Supplementary figures and images for: A Meta-Analysis of Red Yeast Rice: An Effective and Relatively Safe Alternative Approach for Dyslipidemia
Source: PLoS One. 2014 Jun 4;9(6):e98611. doi: 10.1371/journal.pone.0098611 (PMC4045580; doi:10.1371/journal.pone.0098611)

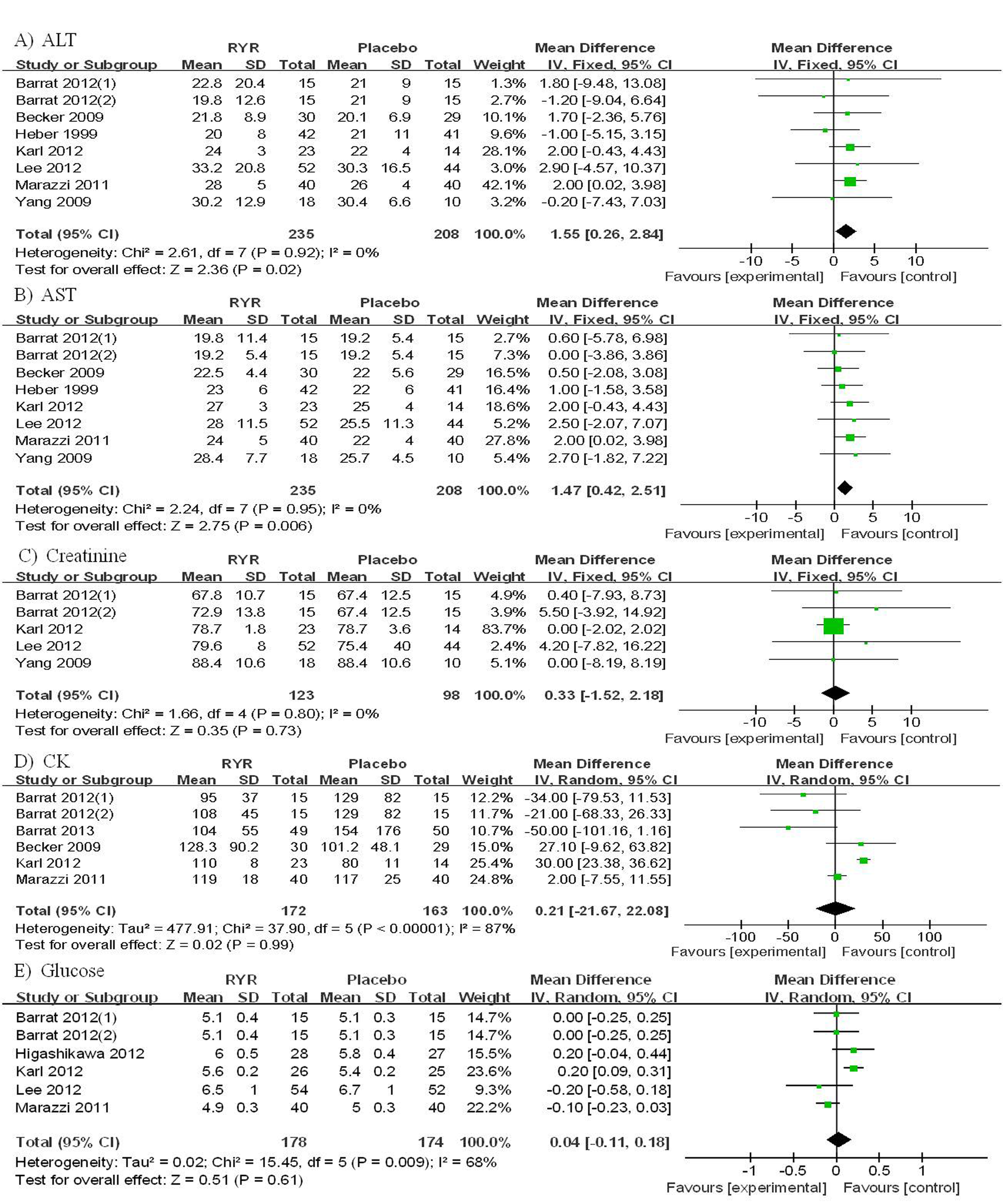

Supplement: Figure S1 — Analysis of secondary outcomes. Abbreviations: alanine transaminase (ALT), aspartate aminotransferase (AST), creatine kinase (CK). (TIF) [file pone.0098611.s001.tif]

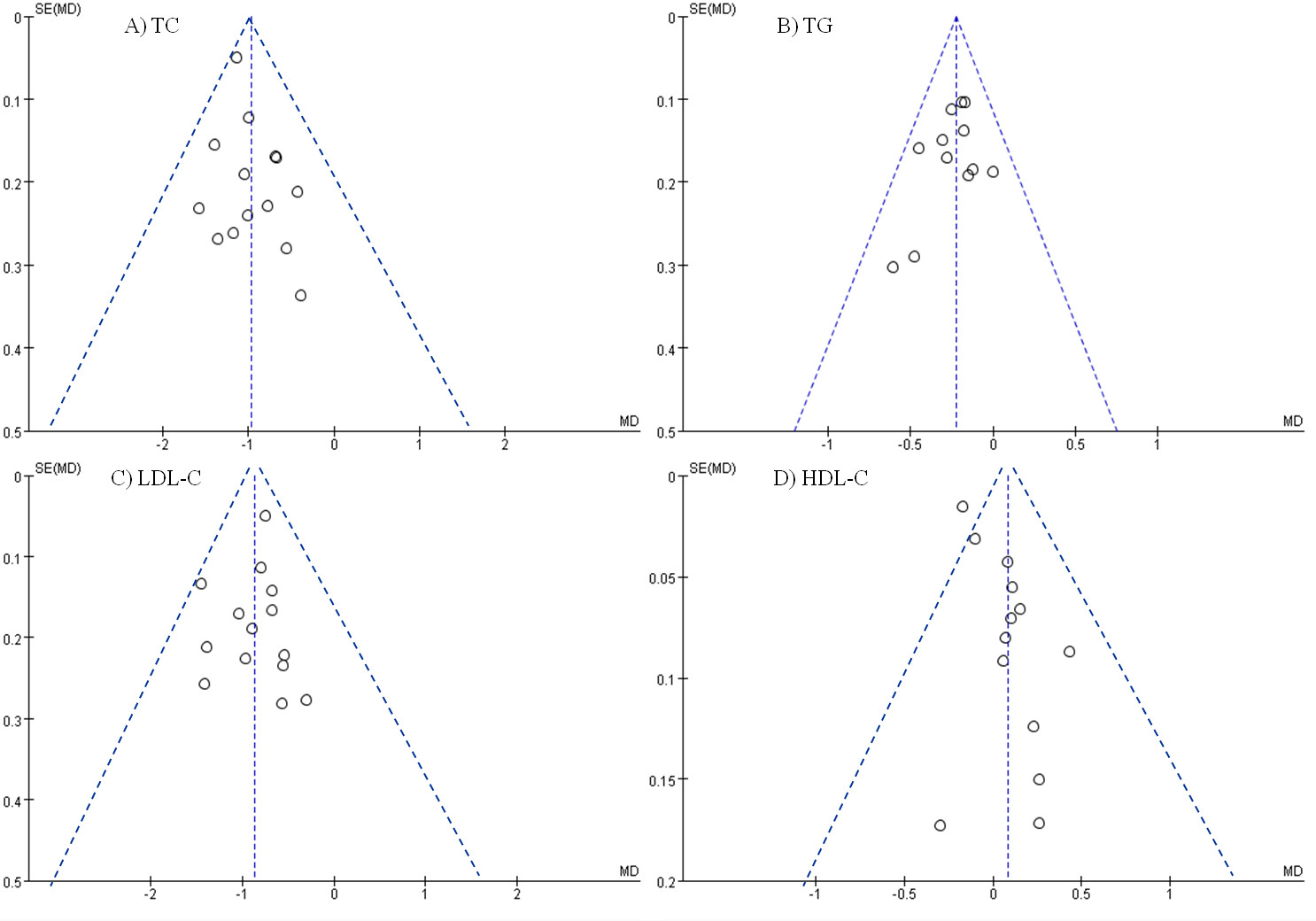

Supplement: Figure S2 — The funnel plots of trials included in the meta-analysis on the effect of Red yeast rice on serum TC (A), TG (B), LDL-C (C), HDL-C (D). Abbreviations: total cholesterol (TC), triglycerides (TG), low-density lipoprotein cholesterol (LDL-C), and high-density lipoprotein cholesterol (HDL-C). (TIF) [file pone.0098611.s002.tif]
