# Supplementary material for: A Meta-Analysis of Red Yeast Rice: An Effective and Relatively Safe Alternative Approach for Dyslipidemia
Source: PLoS One. 2014 Jun 4;9(6):e98611. doi: 10.1371/journal.pone.0098611 (PMC4045580; doi:10.1371/journal.pone.0098611)
Supplement: Table S1 — Quality assessment of included trials (Cochrane risk of bias tool). (DOC) [file pone.0098611.s003.doc]

**Table S2. Quality assessment of included trials (Cochrane risk of bias tool)**

| Reference | Random sequence generation (selection bias) | Allocation concealment (selection bias) | Blinding of participants and personnel (performance bias) | Blinding of assessment (detection bias) | Incomplete outcome data (attrition bias) | Selective reporting (reporting bias) | Other sources of bias |
| --- | --- | --- | --- | --- | --- | --- | --- |
| Ogier 2013 | unclear | unclear | low | low | low | low | low |
| Barrat 2013 | low | unclear | low | low | low | low | low |
| Barrat 2012 | low | unclear | low | low | low | low | low |
| Lee 2012 | unclear | unclear | low | low | low | low | unclear |
| Karl 2012 | low | unclear | low | low | low | low | low |
| Higashikawa 2012 | low | unclear | low | low | low | low | low |
| Marazzi 2011 | unclear | unclear | high | high | low | low | low |
| Bogsrud 2010 | unclear | unclear | low | low | low | low | low |
| Affuso 2010 | unclear | unclear | low | low | low | low | low |
| Yang 2009 | unclear | unclear | low | low | low | low | low |
| Becker 2009 | low | unclear | low | low | low | low | low |
| Huang 2007 | unclear | unclear | high | high | low | unclear | unclear |
| Heber 1999 | unclear | unclear | unclear | unclear | low | unclear | low |

# Low: low risk of bias, high: high risk of bias, unclear: unclear risk of bias
